# Residual quantification and oxidative stress induced by malachite green after subacute and sublethal exposure in red tilapia

**DOI:** 10.14202/vetworld.2019.1416-1421

**Published:** 2019-09

**Authors:** Penz Penz Kwan, Sanjoy Banerjee, Mohamed Shariff, Fatimah Md. Yusoff

**Affiliations:** 1 Laboratory of Marine Biotechnology, Institute of Bioscience, Universiti Putra Malaysia, 43400 UPM Serdang, Malaysia; 2 Department of Veterinary Clinical Studies, Faculty of Veterinary Medicine, Universiti Putra Malaysia, 43400 UPM Serdang, Malaysia; 3 Department of Aquaculture, Faculty of Agriculture, Universiti Putra Malaysia, 43400 UPM Serdang, Malaysia

**Keywords:** malachite green, oxidative stress, red tilapia, subacute, sublethal

## Abstract

**Background and Aim:**

Malachite green (MG) is an effective antiparasitic and antifungal chemical for treatment of fish. However, MG is reported to be a potential carcinogen. Yet, it is widely used in aquaculture despite its prohibition for use in food-producing animals by the EU and USFDA. The present study quantified MG residues and evaluated the oxidative stress in red tilapia when exposed to subacute and sublethal concentrations of MG.

**Materials and Methods:**

Red tilapia exposed to subacute (0.105 mg/L for 20 days) and sublethal (0.053 mg/L for 60 days) concentrations were evaluated for total plasma protein, total immunoglobulin, nitroblue tetrazolium activity, malondialdehyde, reduced glutathione (GSH), and catalase (CAT) activity levels. The residues of MG and leuco-MG (LMG) were also quantified in the fish muscles using liquid chromatography–tandem mass spectrometry.

**Results:**

Fish exposed to subacute concentration showed higher CAT on day 10 in the liver and days 5 and 15 in the spleen, whereas in fish exposed to the sublethal concentration, higher levels of GSH were observed on day 1 in the kidney and day 50 in the spleen. Fish muscle was able to accumulate the sum of MG and LMG of 108.04 µg/kg for subacute (day 20) and 82.68 µg/kg for sublethal (day 60).

**Conclusion:**

This study showed that red tilapia was able to adapt to the stress caused by exposure to MG at sublethal concentration.

## Introduction

Malachite green (MG), a cationic triphenylmethane dye is used as a fungicide and ectoparasiticide in the aquaculture industry since the 1930s [1]. However, the use of MG as a veterinary drug in fish intended for human consumption is not approved by the EU and US Food and Drug Administration, due to its potential carcinogenic, teratogenic, mutagenic, and genotoxic properties [2]. Yet, reports from Rapid Alert System for Food and Feed of the EU in the year 2017 reported detection of MG in many fish species around the world [3], indicating that this chemical is still used by farmers. In this study, red tilapia was selected because it is one of the highly consumed fish in Asia and the Pacific [4]. In addition, tilapia is known for its adaptability to survive in a wide range of environmental conditions [5] as well as in the presence of various pollutants [6,7] such as cadmium [8] and ammonia [9]. MG is highly toxic to mammalian cells and its toxicity is dose [10] and temperature-dependent [1,11].

However, no studies using low dosage of MG have been conducted in fish to evaluate the oxidative stress along with its residue accumulation under tropical conditions where the temperature is relatively high throughout the year.

This study aimed to illustrate the effects of MG and its metabolite leuco-MG (LMG) residues on biochemical profiles and oxidative biomarkers along with the quantification of its residues in red tilapia (*Oreochromis* hybrid) on exposure to subacute and sublethal concentrations of MG.

## Materials and Methods

### Ethical approval

The study was conducted according to animal ethics for research (UPM/IACUC/AUP-R062/2017) of Universiti Putra Malaysia, Malaysia.

### Study animals

A total of 450 juvenile red tilapia (weight 21.5 ± 4.7 g; total length 10.7 ± 0.7 cm) were sourced from a local farm. They were acclimatized to the hatchery conditions for 15 days under natural photoperiod and ambient temperature. Fish were fed with commercial feed (32% protein, Dindings, Malaysia) *ad libitum* twice a day. Water quality parameters such as water temperature, dissolved oxygen, and pH levels ranged between 29.5-30.2°C, 4.7-5.3 mg/L, and 7.2-7.4, respectively, throughout the experiment.

### Experimental design

In this study, red tilapia was exposed to zinc-free MG (Aizen Chemical Co. Ltd., Japan) at subacute (20 days) and sublethal (60 days) concentrations, following the experimental design by Srivastava [12]. There were two treatments and one control with triplicates for each group. There were a total of nine 200 L glass aquarium each containing 50 fish. The subacute (0.105 mg/L) and sublethal (0.053 mg/L) concentrations of MG were 1/10^th^ and 1/20^th^ of the 96 h LC_50_ (1.06 mg/L). No mortalities were observed throughout the experiment. The water and test solutions were renewed daily. Four fish from each treatment and control groups were collected on the 1^st^, 5^th^, 10^th^, 15^th^, and 20^th^ days for subacute and on the 1^st^, 10^th^, 20^th^, 30^th^, 40^th^, 50^th^, and 60^th^ days for sublethal. Fish were anesthetized with tricaine methanesulfonate (Western Chemical, USA) at 100 mg/L and blood samples were taken to measure the nitroblue tetrazolium (NBT) activity [13], total plasma protein (TPP) (Cobas, USA), and total immunoglobulin (TI) levels [13]. Fish were then euthanized to obtain tissue samples of liver, kidney, and spleen to measure oxidative stress and antioxidant levels which included the malondialdehyde (MDA) [14], reduced glutathione (GSH) [15], and catalase (CAT) activity [16] using a spectrophotometer (Ultraviolet 1601 Shimadzu, Japan). Treatment fish were sampled every 5 days for subacute and every 10 days for sublethal concentration to monitor the amount of MG and LMG accumulation in the fish muscle tissue using liquid chromatography–tandem mass spectrometry. The method for sample preparation and chromatography conditions was performed according to Kwan *et al*. [17].

### Statistical analysis

The data were analyzed for statistical significance using repeated measures analysis of variance and Bonferroni *post hoc* test at 95% confidence interval using SPSS Version 21 (IBM, USA).

## Results and Discussion

**Figure-1:**
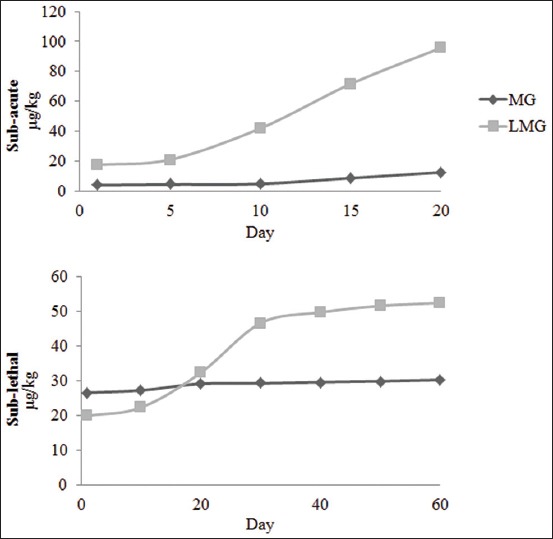
Malachite green (MG) and leuco-MG (LMG) residues in muscle tissue of red tilapia (Oreochromis hybrid) on different days of exposure to subacute and sublethal concentrations of MG.

The method used to quantify the amount of MG and LMG residues achieved CCα of 0.05 µg/L and 0.05 µg/L for MG and LMG, whereas CCβ was 0.09 µg/L and 0.08 µg/L, respectively. From the results (Figure-1), the levels of LMG on average were higher than MG in both subacute and sublethal concentrations. In fish exposed to subacute concentration, levels of MG F(4,4)=79260.228, p=0.002 and LMG F(4,4)=15835.675, p=0.005 increased exponentially according to the days of exposure and reached 12.37 µg/kg of MG and 95.66 µg/kg of LMG in the muscle tissue on day 20. At the sublethal concentration, there was a significant difference between the days of exposure and MG F(6,6)=524.212, p=0.028 and LMG F(6,6)=7972.837, p=0.000 accumulation in the fish muscle tissue. The muscle tissue was able to accumulate up to 30.26 µg/kg MG and 52.42 µg/ kg LMG on day 60. Persistence of MG and LMG in fish is dependent on the concentration and duration of MG exposure as reported by EFSA CONTAM [18]. However, in the present study, a higher amount of residues accumulated in the muscle tissue of fish when exposed to lower concentration of MG as seen on day 1 where the total of MG and LMG residues was 21.12 µg/kg for subacute and 46.48 µg/kg for sublethal exposure (Figure-1).

**Figure-2:**
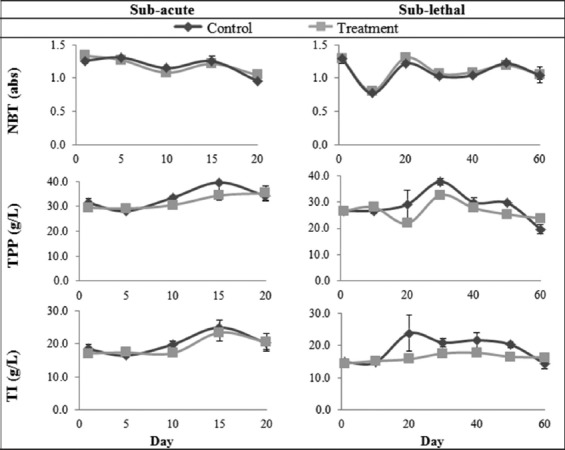
Nitroblue tetrazolium, total plasma protein, and total immunoglobulin in red tilapia (Oreochromis hybrid) exposed to subacute and sublethal concentrations of malachite green. Values with an asterisk (*) indicate statistically significant difference at p<0.05.

The NBT levels in this study showed significant main effect between days of treatment for the subacute F(9,18)=30.99, p=0.031 and sublethal F(13,26)=30.972, p=0.031 concentrations. However, the Bonferroni *post hoc* test did not show any significant difference (p>0.05) between the control and treatment groups (Figure-2). In contrast, Yonar and Yonar [19] reported that the NBT levels in the treatment group were significantly lower than the control group in rainbow trout (*Oncorhynchus mykiss*) exposed to MG for 5 days at 66.67 mg/L and 6.67 mg/L for 30 s and 60 min, respectively. The lower level of NBT reported in rainbow trout compared to the present study could be due to the difference in fish species and duration of exposure in this experiment. Tilapia is more tolerant to cypermethrin when compared to carp (*Cyprinus carpio*), brown trout (*Salmo trutta*), rainbow trout (*Salmo gairdneri*), and rudd (*Scardinius erythrophthalmus*) [20,21]. Hence, the red tilapia may have a higher tolerance toward MG, and therefore, no significant difference in the oxidative radical production of neutrophils between the control and treatment groups was observed in the present study.

The TPP values in the present study for subacute F(9,18)=3.109, p=0.174 and sublethal F(13,26)= 6.783, p=0.097 concentration showed no significant effect between treatment days as well as control and treatment groups (Figure-2). Abdou and Zaky [22] reported that catfish (*Clarias gariepinus*) showed a decrease in total protein on the 3^rd^, 4^th^, and 5^th^ weeks post-exposure to 0.1 mg/L MG. In addition, Srivastava *et al*. [12] reported hypoproteinemia in the treatment group of catfish (*Heteropneustes fossilis*) in the acute (0.20 mg/L for 96 h) as well as short-term exposure (0.10 mg/L and 0.05 mg/L for 10-20 days) to MG. The researchers stated that hypoproteinemia may be due to reduced protein synthesis or protein breakdown caused by kidney disorder (nephrosis) as a consequence of toxicity of MG. In addition, the decrease in protein levels might be associated with the formation of lipoproteins, which are used in the repair of the damaged cell and tissue [23,24]. However, in this study, MG did not affect the TPP levels in red tilapia at subacute and sublethal concentrations.

For the TI, no significant difference was observed for subacute F(9,18)=2.603, p=0.211 and sublethal F(13,26)=3.134, p=0.199 concentrations in this study between control and treatment groups (Figure-2). However, other studies reported a decrease in immunoglobulin levels due to the breakdown of B-lymphocyte caused by MG suppression in rainbow trout exposed to 66.67 mg/L and 6.67 mg/L MG for 30 s and 60 min respectively for 5 days [19]. The blood parameters of red tilapia in this study were not shown to be affected by the subacute and sublethal concentration of MG.

In the present study, red tilapia exposed to subacute concentrations of MG showed no significant effect on the MDA levels in the liver F(9,18)=3.179, p=0.217 and spleen F(9,18)=17.753, p=0.052. There was a significant main effect on the MDA levels in the kidney F(9,18)=24.578, p=0.038. However, the Bonferroni *post hoc* test did not show any significant difference between days. For the sublethal concentration, no significant effect on the MDA levels was observed in the liver F(13,26)=4.541, p=0.167, spleen F(13,26)=14.493, p=0.063, and kidney F(13,26)=6.503, p=0.125. Yet, other studies reported elevated MDA levels in rainbow trout exposed to 5 days bath of MG at 66.67 mg/L and 6.67 mg/L for 30 s and 60 min, respectively [19]. An increase in lipid peroxidation was also reported in *Channa striata* kidney and gill cell lines exposed to 0.1, 1, and 10 µg/mL of MG for 48 h [25]. In both studies, the researchers suggested that MG affected the lipoprotein structure of cell membranes in rainbow trout and *C. striata*.

**Figure-3:**
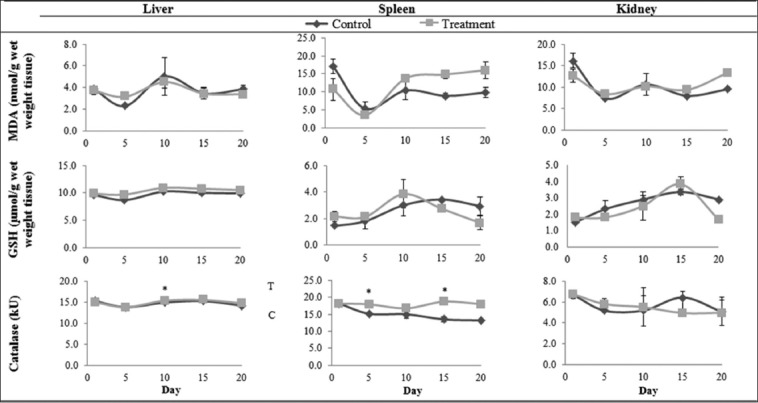
Catalase, reduced glutathione, and malondialdehyde levels in the liver, spleen, and kidney tissues of red tilapia (*Oreochromis* hybrid) exposed to subacute (20 days) concentration of malachite green. Values with an asterisk (*) indicate statistically significant difference at p<0.05.

**Figure-4:**
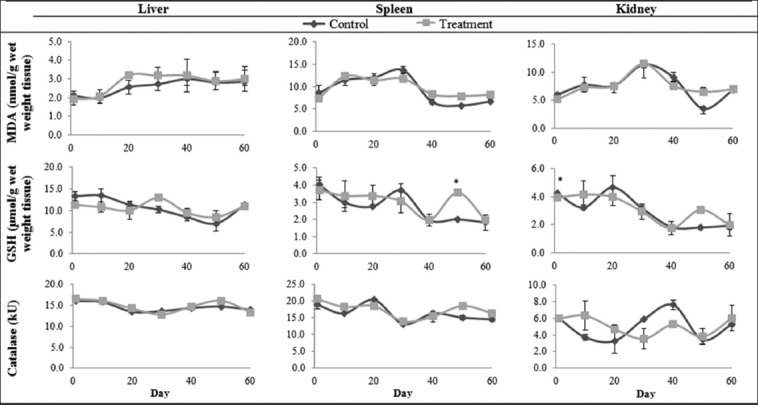
Catalase, reduced glutathione, and malondialdehyde levels in the liver, spleen, and kidney tissues of red tilapia (*Oreochromis* hybrid) exposed to sublethal (60 days) concentration of malachite green. Values with an asterisk (*) indicate statistically significant difference at p<0.05.

The levels of GSH measured in red tilapia exposed to subacute concentration in the present study (Figure-3) showed no significant effect on the liver F(9,18)=17.150, p=0.054, spleen F(9,18)=8.169, p=0.104, and kidney F(9,18)=17.962, p=0.051. Similarly, red tilapia exposed to sublethal concentration (Figure-4) showed no significant effect on the liver F(13,26)=10.536, p=0.083, spleen F(13,26)=8.734, p=0.071, and kidney F(13,26)=12.577, p=0.071. However, the Bonferroni *post hoc* test showed a significant difference between the control and treatment groups on day 1 in the kidney and day 50 in the spleen. According to Atif *et al*. [26], increase in GSH levels is a protective mechanism that fish adopt in the initial phase of exposure to aquatic pollutants. Similarly, this may be the reason for the increase in GSH levels in fish exposed to sublethal concentration. The lack of significant effects in this study suggests that MG exposure at subacute and sublethal concentration were in the tolerable range for red tilapia up to day 40. Yet, higher levels (p<0.05) of GSH were observed in the spleen on day 50, suggesting that red tilapia adopted an important protective mechanism at the later stage of exposure. Higher levels of GSH on day 50 might indicate that there was an increased production of GSH to neutralize free radicals. In addition, it could be a sign of oxidative stress that resulted from an over accumulation of reactive oxygen species. Ahmad *et al*. [27] reported that GSH was used to stabilize oxidative stress by preventing redox cycling.

The CAT level in the liver F(9,18)=22.623, p=0.041 and spleen F(9,18)=27.281, p=0.035 of red tilapia exposed to subacute concentration of MG (Figure-3) showed significant main effects. The Bonferroni *post hoc* test showed a significant difference between the control and treatment group on day 10 in the liver and on day 5 and 15 in the spleen. However, no effects were observed in the kidney F(9,18)=1.572, p=0.337. Similarly, in red tilapia exposed to sublethal concentration, the CAT level in the liver F(13,26)=216.110, p=0.005 and spleen F(13,26)=43.578, p=0.022 showed significant main effects. However, no significant effects were observed in the kidney F(13,26)=6.236, p=0.130. The increased CAT indicates oxidative stress and may have induced a greater activity of the enzyme. However, in another study, a decrease in CAT was reported in the blood, liver, kidney, spleen, and gill of rainbow trout when exposed to 66.67 mg/L and 6.67 mg/L MG at 30 s and 60 min, respectively, for 5 days [19]. A decline in CAT was also reported in the kidney and gill cell line of *C. striata* when exposed to 1 and 10 µg/mL MG [25]. In the present study, the increase in CAT levels may probably be due to an increase in biosynthesis of the enzyme caused by H_2_O_2_ and other free radicals. The different results obtained in the present study as compared to other studies may be due to the different concentrations of MG, exposure period, and fish species.

From this study, the measured parameters for oxidative stress showed significant results on day 15 for the subacute and day 50 for sublethal concentration. On both of these dates, the sum of residues of MG and LMG accumulated for subacute (day 15) was 80.05 µg/kg and sublethal (day 50) was 81.44 µg/kg. Therefore, the toxicity effects of MG were observed when the amount of MG and LMG residues reaches ≥80.05 µg/ kg in the fish muscle. This study showed more significant effects in the subacute concentration because the amount of MG was higher compared to the sublethal concentration. However, at a bigger picture, there were not many significant effects observed between the control and treatment groups in the sublethal concentration. Possibly, red tilapia is a hardy species and might have developed a tolerance and adaptation toward MG at a lower concentration. The previous studies suggested that aquatic organisms are able to develop mechanisms that protect them from pollutants and toxins in the environment. For instance, Atlantic killifish are able to adapt to altered habitat to survive [28].

## Conclusion

This is the first study to indicate the immunological effects of MG in red tilapia along with the residual quantification. The present study concludes that the effects of MG were different based on the concentration of MG and time of exposure. The dosage of toxicant plays an important role in evaluating the effects of toxicity on the fish. Since red tilapia (a hardy species) showed immunological effects when exposed to MG, other fish species that are not as hardy would probably be more susceptible to the toxic effects.

## Authors’ Contributions

PPK performed the study and drafted the manuscript under the supervision of MS, SB, and FMY. MS and SB corrected the manuscript. All the authors have read and approved the final version.
